# Supplementary material for: Development of a novel nanoflow liquid chromatography-parallel reaction monitoring mass spectrometry-based method for quantification of angiotensin peptides in HUVEC cultures
Source: PeerJ. 2020 Sep 15;8:e9941. doi: 10.7717/peerj.9941 (PMC7500351; doi:10.7717/peerj.9941)
Supplement: Data S1 [file peerj-08-9941-s001.pdf]

Supplementary data

Methods

Reagents

Phenol red-free endothelial cell basal medium (ECM-prf, Sciencell Research Laboratories, Carlsbad, CA, USA), endothelial cell growth supplements (ECGS, Sciencell Research Laboratories, Carlsbad, CA, USA); standard samples: Ang I (R&D Systems, Minneapolis, MN, USA), Ang (1-7) (R&D, USA); Ang II (Sigma, St Louis, MO, USA); Ang (1-9), Ang (1-5), Ang III, Ang IV, Val_5-Ang II (internal standard, IS) (Nanjing Peptide Biotechnology Co., Ltd., Nanjing, China; synthesis, purity >98%, mass: 1 mg). Acetonitrile (CAN, LC-MS grade, Fisher Scientific, Hampton, NH, USA), formic acid (FA, LC-MS grade, Fisher Scientific, Hampton, NH, USA), and trifluoroacetic acid (TFA, LC-MS grade, Sigma, St Louis, MO, USA). Experimental water was generated using a Millipore Q ultrapure water meter (Millipore corp., Billerica, MA, USA).

Optimization of the LC-MS/MS conditions and establishment of the PRM methodology

The LC-MS/MS platform (Thermo Fisher Scientific, Waltham, MA, USA) consisted of a preconcentration nanoflow liquid phase system (EASY-nLC 1000 high performance liquid chromatography), capture column (Acclaim Pepmap 100 C18 (100 μm×2 cm, 5 μm, 100 Å)), analytical column (Acclaim Pepmap RSLC C18 (50 μm×15 cm, 2 μm, 100 Å)) combined with nano-electrospray ionization (nano-ESI) and Q Exactive quadrupole orbitrap high-resolution mass spectrometry. The system

was controlled by the Xcalibur software (v. 3.1) and the analysis software for mass spectrometry data was Skyline (v.4.1.0, MacCoss Lab, University of Washington, USA).

Construction of the HUVEC model cell lines with silenced ACE expression

Two shRNA sequences targeting ACE mRNA were designed based on the upstream and downstream sequences of the *ACE* gene in Genbank (number: NM_001178057.1→NP_001171528.1). A non-human homologous shRNA was used as the negative control. Homologous analysis was performed on the target sequence by BLAST to exclude the shRNA non-specific sequences. Ultimately, the shRNA sequences were determined. The 5' end of the coding strand of the above sequence was introduced into the *AgeI* cleavage site.The EcoRI cleavage site was introduced into the 5' end of the template strand. The sequence of the stem loop was 5'-CTCGAG-3'. The ACE targeted gene, shRNA sequences of the negative control gene, and the control sequences are shown in Supplementary Table S1. The above three ACE-shRNA sequences were ligated to the pLKO.1 vector plasmid to form a recombinant plasmid. The DH5α competence was used for the preparation of a large quantity of plasmids.

The 293FT cells were seeded in a 6-well plate and cultured at 37 ℃ in 5% CO_2 to a confluence of 70%-80%. The transfection mixture was prepared and divided into an interference group and a control group. The pLKO.1 recombinant plasmids containing the target sequence (1.6 μl), psPAX2 (0.8 μl), and pCMV-VSV-G (1.2 μl) were mixed with Lipofectamine 3000 at a ratio of 1:1. The mixed solution was added to the 6-well

plate and the virus supernatant was collected after 48 h.

The HUVECs were divided into three groups. One control group, named the pLKO.1 group, was transfected with the lentivirus packaged with the pLKO.1 plasmid of the negative control sequence. The other two groups were the interference groups, and the lentiviruses packaged with the ACE-shRNA-1 and ACE-shRNA-2 interference sequence plasmids were transfected, respectively. The cellular RNA was extracted after puromycin screening to detect the ACE mRNA levels.

Detection of the Ang peptides from HUVECs

For the detection of Ang I and its metabolites, three groups of cells were prepared in T25 flasks and passaged in 6-well plates. Three duplicate wells were set for each group. When the cells were grown to 70% confluence, the ECM-prf culture medium containing 1% ECGS was added to each well and cultured at 37 ℃, 5% CO_2 for 24 h. Then, Ang I at a final concentration of 1 μM was added. Culture was continued for 1 h and 100 μl of supernatant were extracted and added into an ice-pre-cooled low protein attachment LoBind centrifuge tube (1.5 ml, Eppendorf, Hamburg, Germany). Then, 2 μl of mixed protease inhibitor, 2 μl of 50 mM EDTA solution, 2.5 μl of 4% 2-mercaptoethanol, and internal standard Val_5-Ang II 10 ng were added to each tube. The samples were mixed and centrifuged at 4 ℃, 2000 ×g, for 4 min. The samples were processed LC-MS/MS detection.

For the detection of the ACE protein, the 6-well plates were washed with pre-cooled PBS once per well and 100 μl of RIPA buffer (containing 1% PMSF) was

added. After complete lysis, the samples were centrifuged at low temperature and the supernatant was extracted for BCA protein concentration assay. After mixing and boiling with the loading buffer, the protein sample was subjected to SDS-PAGE electrophoresis and transferred to a polyvinylidene fluoride (PVDF) membrane. The membrane was blocked with TBST with 5% nonfat milk for 1 h. The ACE rabbit anti-human monoclonal antibody (1:1000, Abcam, Cambridge, United Kingdom) and β-actin rabbit anti-human monoclonal antibody (1:1000, Cell Signaling, Danvers, MA, USA) were added in the TBST with 5% nonfat milk and incubated at 4 ℃ overnight. The membranes were washed with TBST three times, 15 min each time. The horseradish peroxidase labeled mouse anti-rabbit secondary antibody (1:10,000, Jackson Immuno Research, West Grove, PA, USA) was incubated at room temperature for 2 h and washed with TBST three times, 15 min each time. The membranes were exposed in the darkroom. The Image J software was used to analyze the area and gray scale of the bands.

QRT-PCR detection of the ACE mRNA levels

The RNA extracted from each HUVEC model group was reversely transcribed to cDNA and detected using a real-time quantitative STRATAGENE Mx3000p PCR instrument. The primer sequences are shown in Supplementary Table S2. The 18s rRNA was used as the normalized internal standard and the $2^{-\Delta\Delta CT}$ method was used to calculate the relative expression of ACE mRNA (Supplementary Table S3). The formula for the down-regulation efficiency of ACE mRNA was: ηRNA= (expression

levels of ACE gene in pLKO.1 group- expression levels of ACE gene in ACE-shRNA group) / expression levels of the ACE gene in the pLKO.1 group.

Supplementary Table S1. The plasmid framework sequences of the plasmids carrying the shRNA interfering sequences targeting *ACE* gene and the control sequences

Name	5'	Stem	Loop	Stem	3'
ACE-shRNA-1 For	CCGG	GCTAAGTTTCA TGTTCCAAAT	CTCGAG	ATTTGGAACAT GAAACTTAGC	TTTTTG
ACE-shRNA-1 Rev	AATT CAAAAA	GCTAAGTTTCA TGTTCCAAAT	CTCGAG	ATTTGGAACAT GAAACTTAGC	
ACE-shRNA-2 For	CCGG	CGTGCCTTACA TCAGGTACTT	CTCGAG	AAGTACCTGAT GTAAGGCACG	TTTTTG
ACE-shRNA-2 Rev	AATT CAAAAA	CGTGCCTTACA TCAGGTACTT	CTCGAG	AAGTACCTGAT GTAAGGCACG	
pLKO.1 (NC)	CCGG	CCTAAGGTTAA GTCGCCCTCG	CTCGAG	CGAGGGCGAC TTAACCTTAGG	TTTTTG
pLKO.1 (NC)	AATT CAAAAA	CCTAAGGTTAA GTCGCCCTCG	CTCGAG	CGAGGGCGAC TTAACCTTAGG	

NC was negative control group

Supplementary Table S2. Primer sequences for qRT-PCR

Name	Sequences
ACE For	5’-TGGTGTGGAACGAGTATGC-3’
ACE Rev	5’-AGGGTGTGGTTGGCTATTTG-3’
18s rRNA For	5’-TAGAGGGACAAGTGGCGTTC-3’
18s rRNA Rev	5’-CGCTGAGCCAGTCAGTGT-3’

Supplementary Table S3. Relative expression levels of the ACE and 18s rRNA mRNA in HUVECs transfected with different interference sequences

Samples		ACE	18s	ΔCt	ΔΔCt	RQ
pLKO.1	Ct1	24.68	13.21	11.05	0.46	0.73
	Ct2	24.1	14.15	10.47	-0.12	1.09
	Ct3	23.88	13.52	10.25	-0.34	1.27
ACE-shRNA-1	Ct1	29.32	12.19	18.05	7.45	0.01
	Ct2	28.56	10.81	17.29	6.69	0.01
	Ct3	28.75	10.82	17.48	6.88	0.01
ACE-shRNA-2	Ct1	24.86	12.66	12.68	2.08	0.24
	Ct2	24.82	11.94	12.64	2.04	0.24
	Ct3	24.65	11.95	12.47	1.87	0.27

Note: pLKO.1 (NC) was a blank plasmid group containing no interference sequence as served as negative control.

Supplementary Table S4. Molecular description and PRM settings for LC-MS/MS detection of angiotensin peptides

**Peptides**	**Amino acid sequence**	**MW (Da)**	**Precursor ion (m/z)**	**Charge state**	**Quantitative production (m/z)**
Ang I	DRVYIHPFHL	1295.7	432.9	3+	$b4^+$ 534.3
Ang (1-9)	DRVYIHPFH	1182.6	395.2	3+	$b4^+$ 534.3
Ang II	DRVYIHPF	1045.5	523.8	2+	$b6^+$ 784.4
Ang (1-7)	DRVYIHP	898.5	450.2	2+	$b5^+$ 647.4
Ang (1-5)	DRVYI	664.4	333.2	2+	$b3^+$ 371.2
Ang III	RVYIHPF	930.5	466.3	2+	$b5^+$ 669.4
Ang IV	VYIHPF	774.4	388.2	2+	$y3^+$ 400.2
Val^5-Ang II*	DRVYVHPF	1031.5	516.8	2+	$b6^+$ 770.4

*Internal standard

Supplementary Table S5. Slope and intercept of calibration curves, correlation coefficients, and linear ranges for angiotensin peptides extracted from HUVEC culture supernatant samples.

	Slope	Intercept	R^2	Linear range		LLOQ
				(ng/ml)	(pg on column)	(ng/ml)
Ang I	1.599	-4.6056	0.9897	1-1000	2-2000	1
Ang (1-9)	1.1531	-2.3631	0.9953	0.25-1000	0.5-2000	0.25
Ang II	1.154	-3.7026	0.9945	0.05-1000	0.1-2000	0.05
Ang (1-7)	1.0803	-2.2641	0.9967	0.25-500	0.5-1000	0.25
Ang (1-5)	1.2272	-3.0175	0.9922	0.25-1000	0.5-2000	0.25
Ang III	1.1286	-2.2918	0.9974	0.05-1000	0.1-2000	0.05
Ang IV	1.2389	-3.4251	0.9945	0.05-500	0.1-1000	0.05

Supplementary Table S6. Intra-day and inter-day precisions of angiotensin peptides extracted from HUVEC culture supernatant QC samples

	Concentration (ng/ml)	Relative standard deviation (n=6) (%)		Retention time (min)	
		Intra-day	Inter-day	Intra-day	Inter-day
Ang I	10	4.3	9.8	31.91±0.05	34.96±0.36
Ang (1-9)	10	1.5	6.3	17.66±0.04	20.40±0.35
Ang II	10	2.4	8.6	27.66±0.03	30.28±0.25
Ang (1-7)	10	4.2	8.8	14.36±0.03	16.27±0.21
Ang (1-5)	10	8.7	3.1	17.19±0.03	20.08±0.36
Ang III	10	2.9	7.4	19.05±0.03	21.80±0.44
Ang IV	10	3.5	4.9	29.31±0.07	32.09±0.24

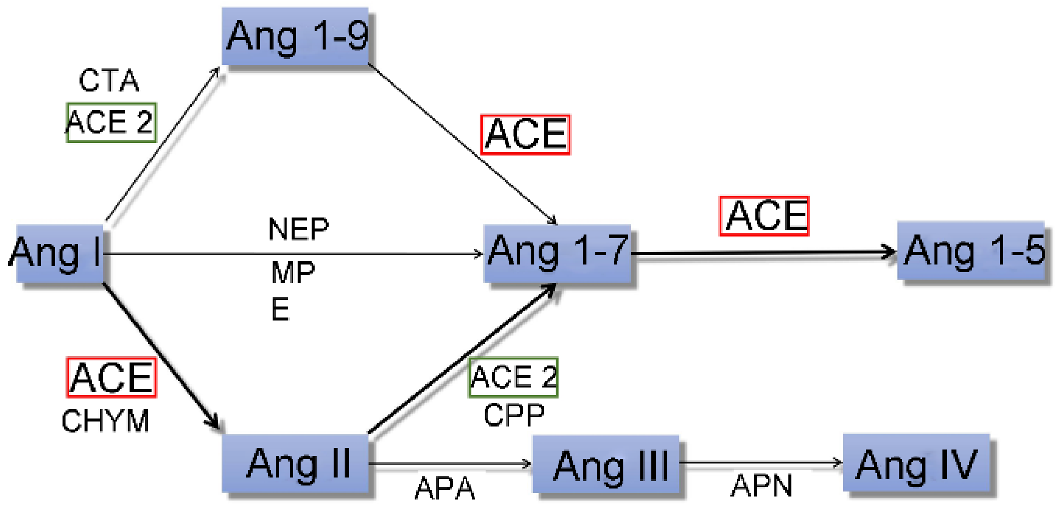

Supplementary Figure S1. The angiotensin I metabolism pathways [1]. ACE: angiotensin converting enzyme; ACE2: angiotensin converting enzyme 2; CTA: cathepsin A; CHYM: chymase; NEP: neutral endopeptidase; MP: metalloprotease; E: endopeptidase; CPP: carboxypeptidase P; APA: aminopeptidase A; APN: aminopeptidase N.

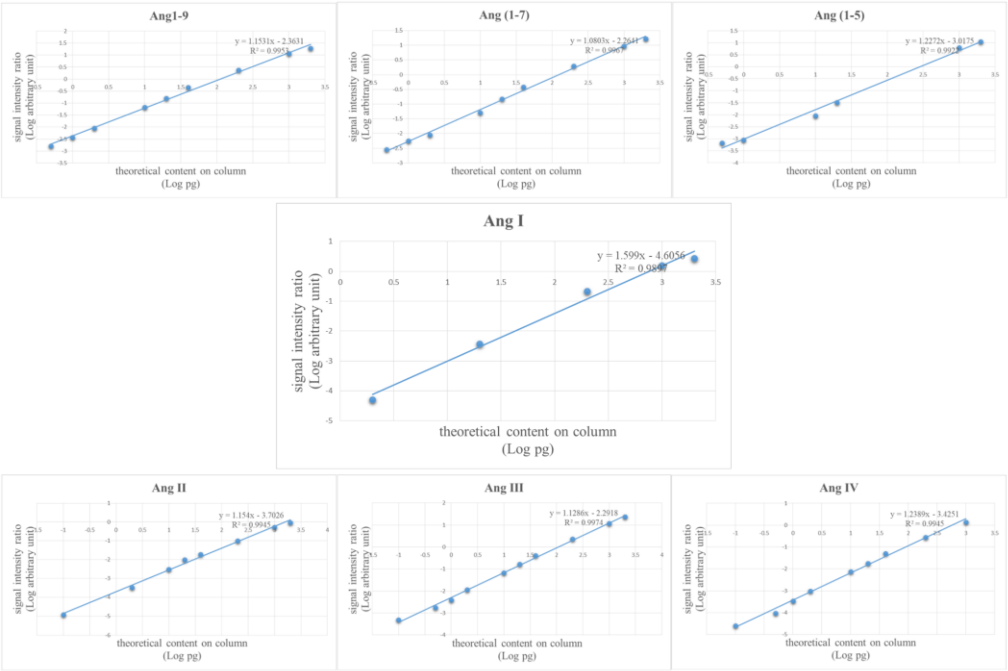

Supplementary Figure S2. Linear correlation coefficients of the standard curves were analyzed by least-squares regressions. All coefficients were ≥0.99.

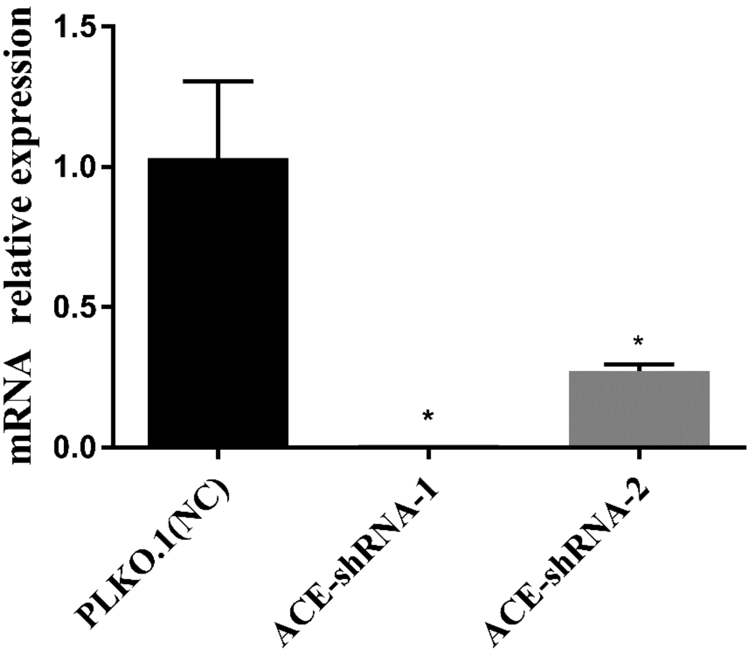

Supplementary Figure S3. ACE mRNA expression in different shRNA interference groups.*P<0.05 vs. the negative control (NC) group. The qRT-PCR results showed that compared with the negative control group transfected with negative sequence plasmid pLKO.1, the expression of ACE mRNA in the two interference groups was significantly reduced (P<0.001). Hereby, the silencing efficiency of *ACE* gene was calculated as follows: 73% in the ACE-shRNA-2 group and 99% in the ACE-shRNA-1 group.

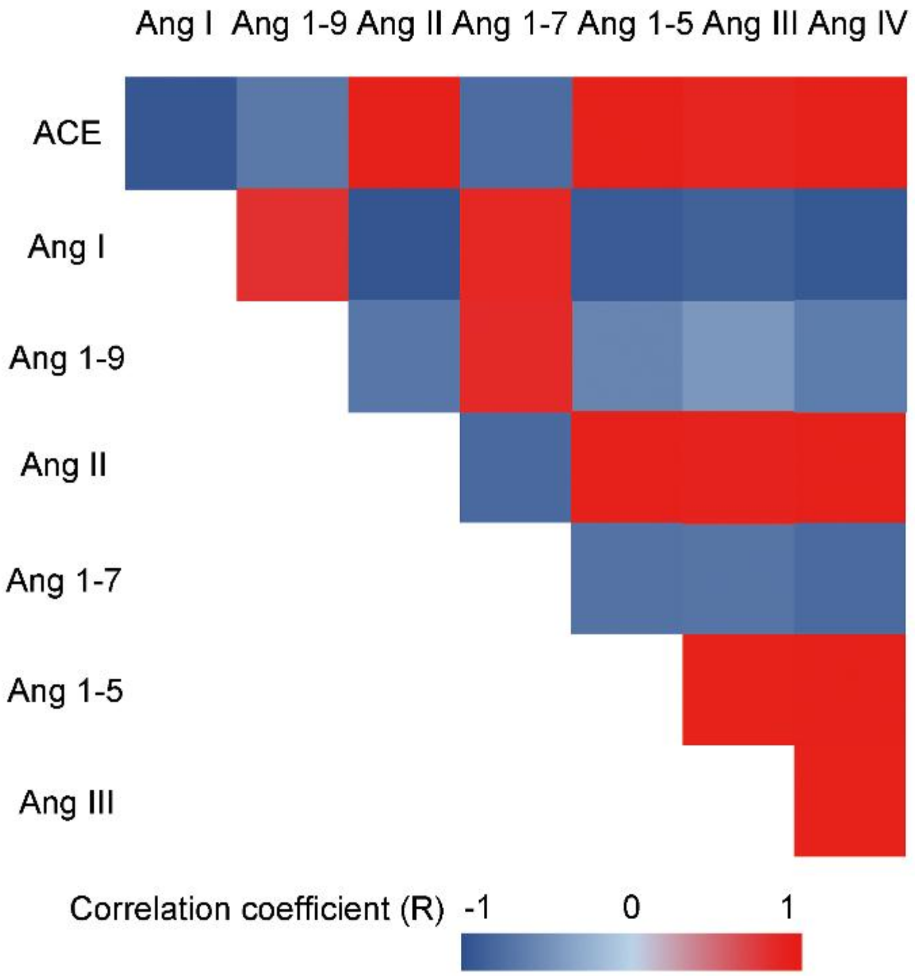

Supplementary Figure S4. Correlations between the concentrations of angiotensin metabolites and angiotensin-converting enzyme expression in HUVECs.

REFERENCES

1. Bujak-Gizycka B, Madej J, Wolkow PP, Olszanecki R, Drabik L, Rutowski J, et al. (2007) Measurement of angiotensin metabolites in organ bath and cell culture experiments by liquid chromatography - electrospray ionization - mass spectrometry (LC-ESI-MS). J Physiol Pharmacol 58: 529-540.
