# Supplementary figures and images for: Development of a novel nanoflow liquid chromatography-parallel reaction monitoring mass spectrometry-based method for quantification of angiotensin peptides in HUVEC cultures
Source: PeerJ. 2020 Sep 15;8:e9941. doi: 10.7717/peerj.9941 (PMC7500351; doi:10.7717/peerj.9941)

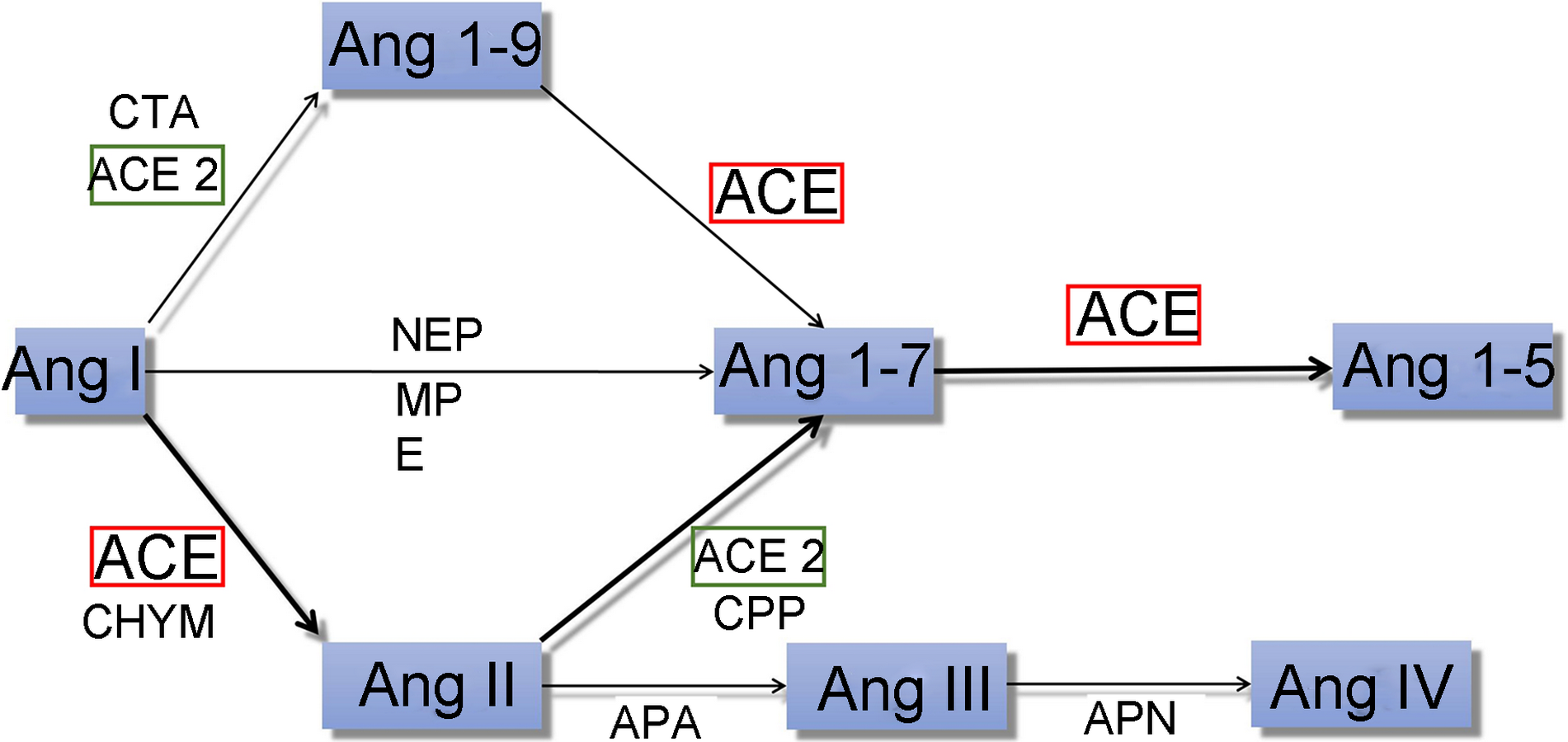

Supplement: Figure S1 — ACE: angiotensin converting enzyme; ACE2: angiotensin converting enzyme 2; CTA: cathepsin A; CHYM: chymase; NEP: neutral endopeptidase; MP: metalloprotease; E: endopeptidase; CPP: carboxypeptidase P; APA: aminopeptidase A; APN: aminopeptidase N. [file peerj-08-9941-s002.png]

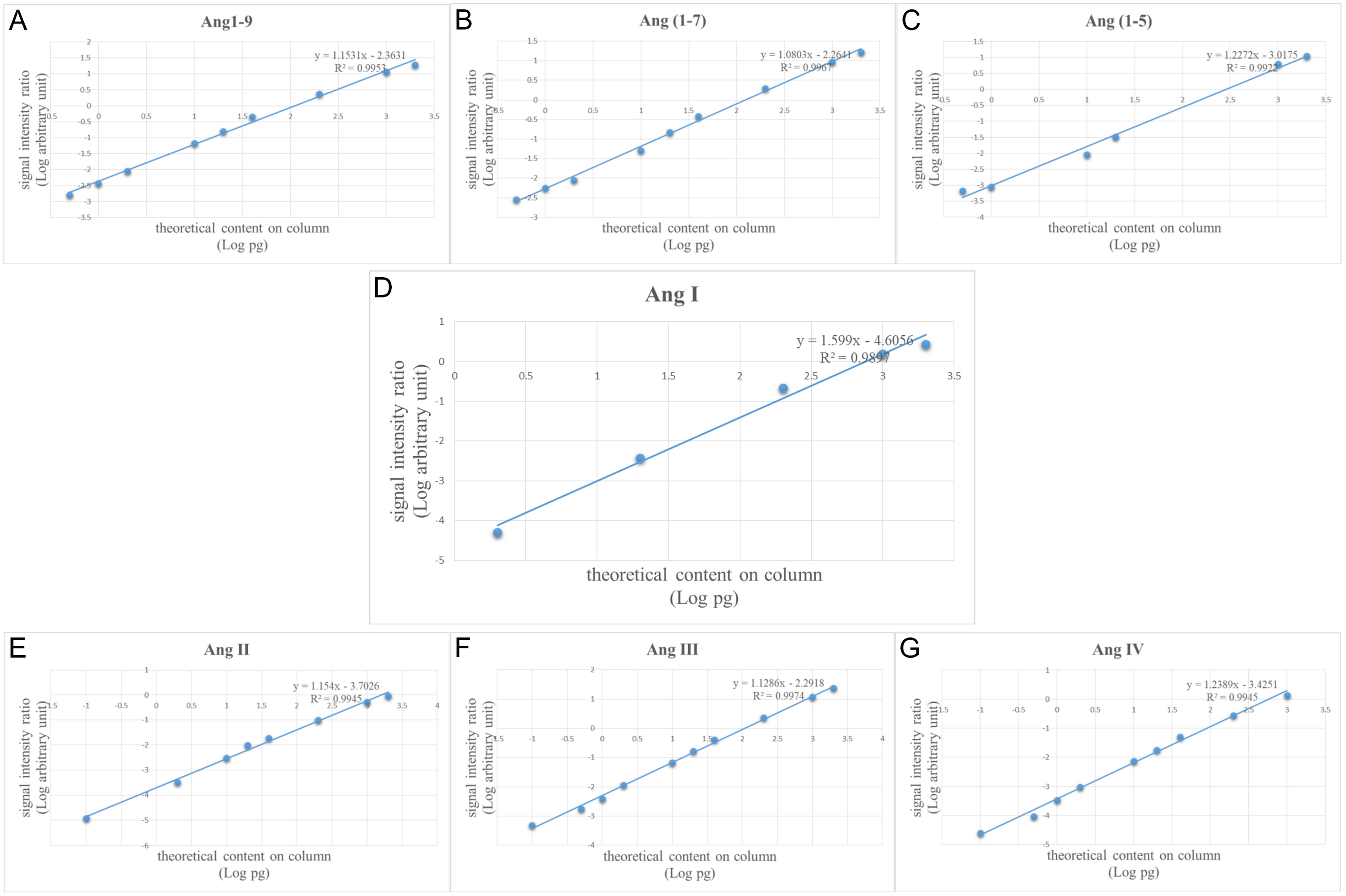

Supplement: Figure S2 — All coefficients were ≥0.99. [file peerj-08-9941-s003.png]

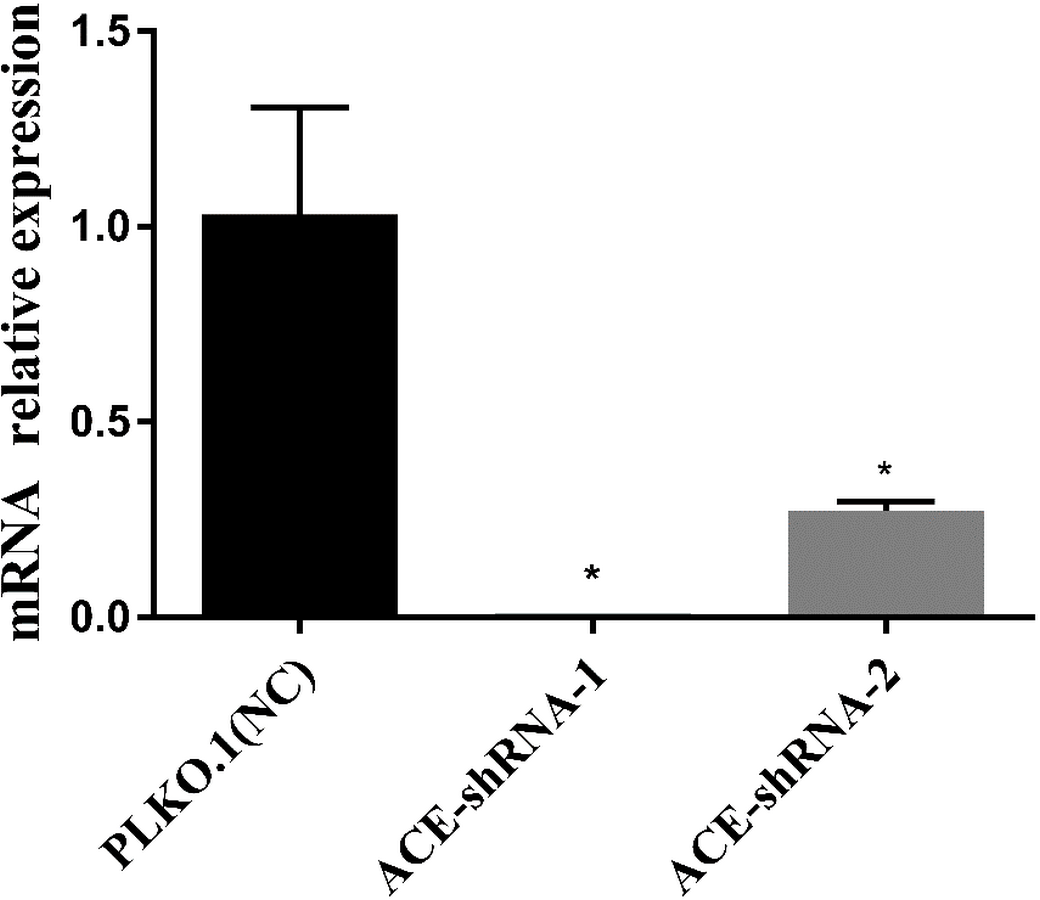

Supplement: Figure S3 — *P¡0.05 vs. the negative control (NC) group. The qRT-PCR results showed that compared with the negative control group transfected with negative sequence plasmid pLKO.1, the expression of ACE mRNA in the two interference groups was significantly reduced (P < 0.001). Hereby, the silencing efficiency of ACE gene was calculated as follows: 73% in the ACE-shRNA-2 group and 99% in the ACE-shRNA-1 group. [file peerj-08-9941-s004.png]

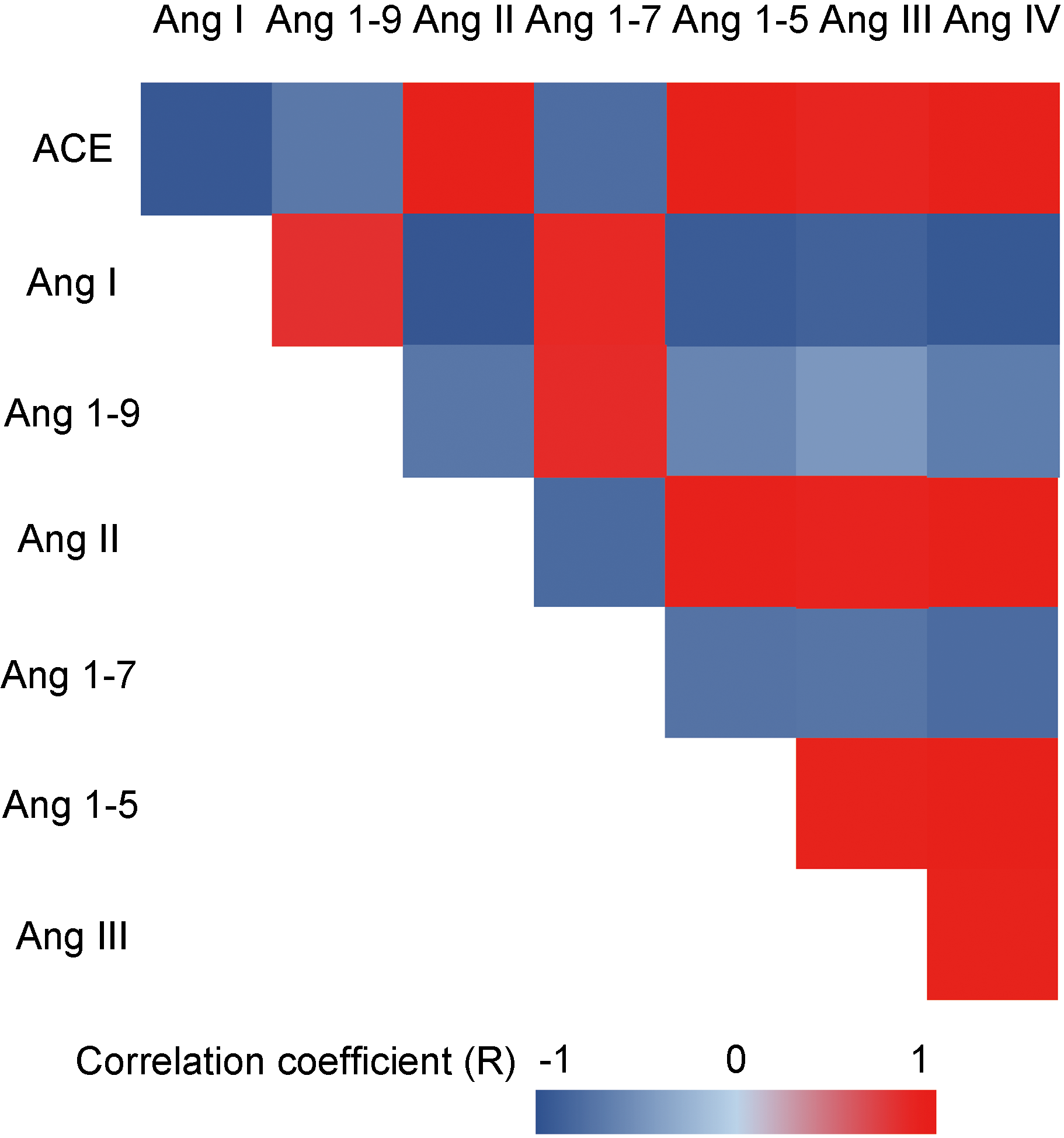

Supplement: Figure S4 [file peerj-08-9941-s005.png]

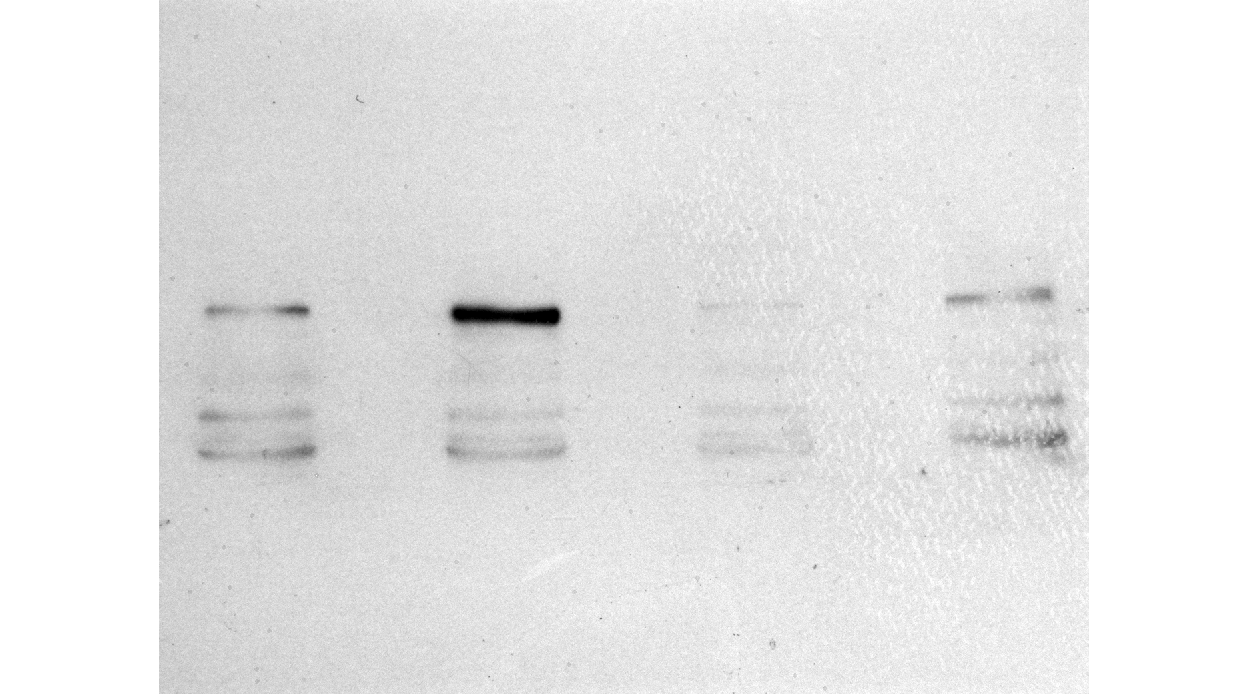

Supplement: Supplemental Information 7 — This is the full-length uncropped blots, the far left lane is not relevant to this experiment. [file peerj-08-9941-s007.bmp]

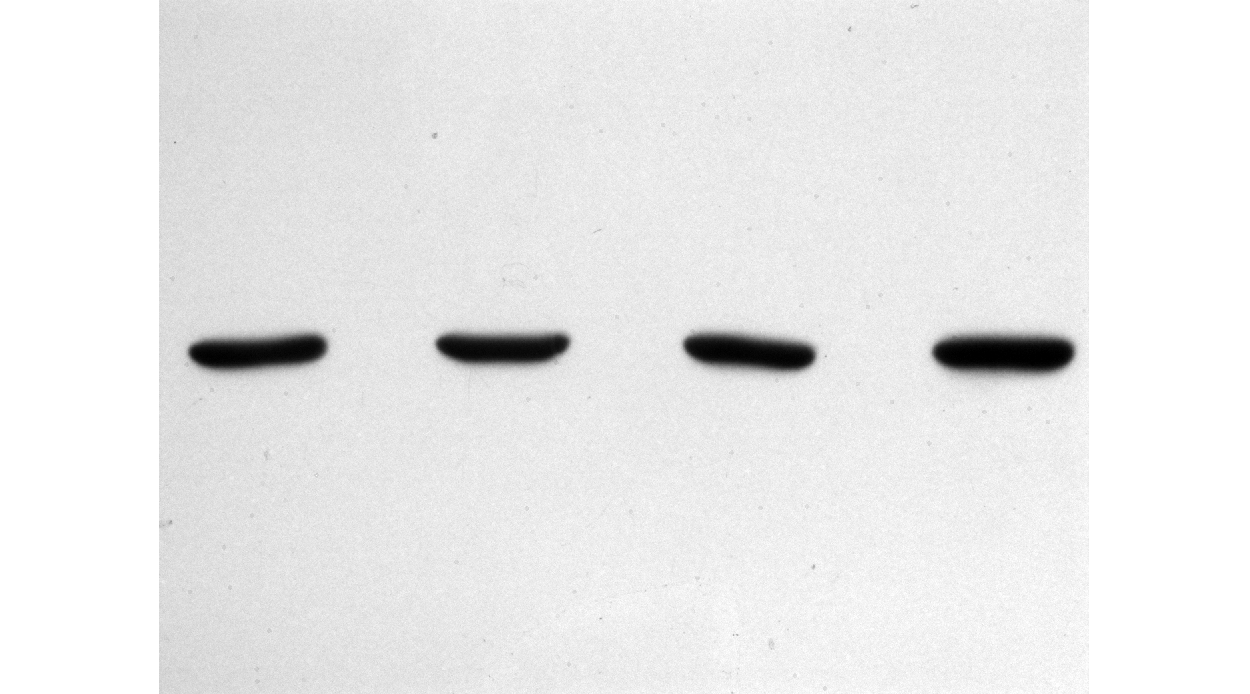

Supplement: Supplemental Information 8 — This is the full-length uncropped blots, the far left lane is not relevant to this experiment. [file peerj-08-9941-s008.bmp]
